# Assessment of the Relationship between Periodontitis and Cardiac Parameters in Patients with Early Chronic Heart Failure: A Cross-Sectional Study

**DOI:** 10.3390/jfmk9010052

**Published:** 2024-03-18

**Authors:** Antina Schulze, Stefan Kwast, Christoph Pökel, Martin Busse

**Affiliations:** 1 General Outpatient Clinic of Sports Medicine, University of Leipzig, 04103 Leipzig, Germany; christoph.poekel@uni-leipzig.de (C.P.); drmartinbusse@gmail.com (M.B.); 2 Helios Health Institute GmbH, 13125 Berlin, Germany; stefan.kwast@helios-health-institute.com

**Keywords:** heart failure, periodontitis, troponin, NT-proBNP, exercise performance, incremental testing, oral health

## Abstract

Periodontal disease (PD) is considered a risk factor for cardiovascular events. However, its relationship to chronic heart failure (CHF) is unclear. The aim was to compare cardiac and inflammatory parameters in CHF patients with (PG) versus without periodontitis (NPG). The following parameters were recorded in 58 patients: periodontal screening and recording (PSR), troponin T, NT-proBNP, C-reactive protein (CRP), interleukin-6 (IL-6), blood pressure, heart rate, ejection fraction (EF), ventricular systolic and diastolic function parameters, incremental test, and three questionnaires (Mediterranean Diet Adherence Screener, MEDAS; Oral Health Impact Profile, OHIP-14; Patient Health Questionnaire, PHQ). The serum levels of NT-proBNP and troponin T were significantly higher in the PG, and the left ventricular systolic and diastolic function parameters were significantly lower. The correlation analysis showed age as the only independent risk factor for periodontitis and cardiac biomarkers. No significant group differences were found in the MEDAS, OHIP-14, and PHQ scores, or in CRP, IL-6, and cardiocirculatory parameters. Overall, the BMI correlated significantly with the mean PSR and total cholesterol. The occurrence of increased PSR together with increased age and cardiac risk parameters does not exclude an association between periodontitis and CHF, though no positive correlation was calculated. Periodontitis may be a modifiable risk factor for CHF. Its treatment may help to control the inflammatory burden.

## 1. Introduction

Heart failure (CHF) is a pro-inflammatory syndrome with multiorgan involvement [1] and a prevalence of 8% in the population aged > 45 years. Common underlying conditions of CHF include coronary artery disease, arterial hypertension, myocarditis, diabetes mellitus, and systemic inflammation, including periodontitis [2]. Diabetes mellitus is a strong risk factor for the development of CHF; however, there is also evidence for reciprocal effects [3]. Patients with diabetes are up to five times more likely to suffer from heart failure than those with a healthy metabolism [4]. The fact that CHF is closely linked to diabetes is due to an overall metabolic disorder with possible negative effects on arteries and coronary vessels. A long-term elevated blood glucose level increases chronic inflammatory processes in the vessels. The vessel walls become stiff with the effect of an impaired diastolic function [5] and left ventricular concentric remodeling [6]. Conversely, heart failure triggers diabetic processes that increase glucose metabolism and cause insulin resistance [3]. Thus, the inflammatory mechanisms probably contribute directly to the structural myocardial changes and limitation of cardiac function, as well as to the progression of the associated symptoms [2]. Furthermore, genetic causes have been documented. Over the next 25 years, heart failure rates are expected to increase further by approximately 50%. The high number of unreported cases is particularly problematic. This is due to the frequent lack of recognition of the early stages of the disease, where an improvement or even complete regression is most likely to occur [7]. As a syndrome, CHF particularly affects degeneration, exercise tolerance, and causes chronic inflammation of the heart, muscles, and vessels [8]. Previous studies showed bone catabolism in a case of CHF [9,10], which can be linked to periodontal disease.

Several chronic infectious, inflammatory, and immune diseases are associated with significantly higher risks of adverse cardiovascular events [11]. Periodontal disease (PD) is a chronic non-communicable disease with a high prevalence and is the sixth most common human disease [12,13,14]. Risk factors are dental plaque, calculus, age, genetics, smoking, and diabetes [15]. Gingival bleeding and periodontitis are sensitive markers for abnormalities in the macronutrient content (excessive carbohydrate or polyunsaturated fat intake and deficient protein intake) and micronutrient intake too. Nutrition is extremely important and therefore, well placed to deliver general, oral, and dental health. A well-studied way to improve general health is through the Mediterranean diet (MD) [16], which effectively reduces the body weight and risk factors for metabolic syndrome [17,18]. So far, the relationship between CHF, periodontitis, and MD has not been studied. We therefore compared the diets of CHF patients with and without periodontitis.

Periodontitis can trigger systemic inflammation by actuating the acute hepatic phase response owing to the possible bacteremia [19,20]. As low-grade inflammation, PD may represent one of several possible causal factors for heart diseases. Due to its high prevalence in the population, it may contribute a significant part to the infection-associated risk for cardiovascular disease (CVD) [21]. Coronary artery disease is a major cause of CHF. Therefore, an association between periodontitis and CHF seems to be possible. Both have common risk factors [22]. Fröhlich et al. [2] showed a higher prevalence of periodontitis in patients with CHF, but in contrast to Wood and Johnson [23], showed no relationship between the severity of periodontitis and severity of symptoms caused by CHF. Moreover, Schulze-Spate et al. [24] reported that severe periodontitis is related to increased inflammatory mediators and cytokines in patients with CHF. A direct relation between oral hygiene indicators and CHF has also been shown [25]. An independent association between severe periodontitis and cardiovascular disease has also reported by Tonetti and Van Dyke [26]. Proposed mechanisms include bacteremia and the associated systemic inflammatory sequelae, including higher C-reactive protein (CRP) values and oxidative stress [27]. Jockel-Schneider et al. [28] reported a greater arterial stiffness in patients with periodontitis. The increase in arterial stiffness was associated with a left ventricular hypertrophy [29,30]. Systemic inflammation has an important effect on the structure and function of the left ventricle [31]. Therefore, the increased levels of inflammatory markers due to periodontitis may be indirectly related to CHF and structural changes in the heart [32,33,34]. Moreover, the severity of periodontitis is related to left ventricular hypertrophy [32,33]. Aoyama et al. [35] showed a higher prevalence of CHF in patients with higher antibody levels due to periodontitis. These facts altogether lead to the thesis that chronic elevations in the systemic inflammatory burden are causally related to CVD [14] and CHF development. However, the mechanisms are not clear.

Boyapati et al. [19] have reported a positive relation between troponin T levels and the grade of periodontitis. The aim of the current study on this topic was to analyze the instance of periodontitis in patients with CHF, and to deduce a probable association between periodontal disease and CHF by correlating the cardiac biomarkers showing cardiac lesions (hsTropT, NT-proBNP, and hsCRP) and clinical periodontal as well as inflammatory parameters. N-terminal pro b-type natriuretic peptide (NT-proBNP) is most concentrated in the left ventricular myocardium. In the case of heart failure, the left ventricle works harder and produces more BNP and NT-proBNP [36].

Given the potential impact of periodontitis on cardiovascular health and the limited literature on this topic, this study investigated the relationship between periodontitis and CHF. It is the first study attempt to compare and correlate inflammatory serum biomarkers and exercise load parameters in CHF patients with and without periodontitis.

## 2. Materials and Methods

### 2.1. Study Design

The objective of the presented data set refers to a part of the baseline data from the “HITS” study (“Heart failure, Individual exercise training, Telemonitoring, Self-management”) at the study center the Outpatient Clinic of Sports Medicine, University of Leipzig, Germany. The study was registered in 2020 at the German Clinical Trials Register under DRKS00019022 on 28 May. The approval was granted by the medical ethics committee of the University of Leipzig (2019/No. 479/19-ek). HITS is a partially ongoing multicenter, prospective, both therapeutic and diagnostic, and parallel-group study being conducted as a collaborative project between Leipzig University, Hannover Medical School, Leipzig Heart Center, AOK plus health insurance, IGES Institute, Diavention GmbH, and the Clinical Centers in Wolfsburg, Chemnitz, and Dresden (Germany). The HITS innovation fund project aims to establish a new care model including the early diagnosis and treatment of patients with CHF according to the American Heart Association guidelines [37,38]. The NYHA functional classification was used to estimate patients’ functional abilities based on their symptoms [39]. We included both existing CHF patients and newly diagnosed patients. The exclusion criteria were the lack of compliance, alcohol abuse, active participation in other studies, and any physical or mental disabilities.

This manuscript focuses on specific baseline data from the original “HITS” study with a different investigation objective. With the interaction of periodontitis and impaired cardiac function without intervention or randomization, the study describes a subsidiary/subordinated aspect of the main study. The group classification was based on periodontal parameters, which were measured during the baseline examination. The data and the course of the original study are described by others and will be submitted for publication. In addition, further sub-studies on different aspects of the original study are planned.

### 2.2. Participants

After several information events, a total of 131 subjects were assessed for eligibility and periodontally examined at the study center the Institute of Sports Medicine, University of Leipzig. The inclusion criteria for the analysis were a present tooth in ≥5 of the sextants, more than 10 teeth, either no periodontitis or periodontitis with a presence of ≥2 sextants with periodontal screening and recording (PSR) code 4 (probing pocket depth (PPD) of ≥5.5 mm), participation in incremental testing, nonsmokers, older than 18, and up to 80 years old with diagnosed CHF. Exclusion criteria were the lack of compliance, pregnancy, antibiotic therapy less than 3 months prior to the dental examination, alcohol abuse, periodontal treatment in the past 6 months, medically and immunocompromised patients, risk of systemic complications, and autoimmune disorders. Of 131 patients, 58 qualified for the present analysis. The patients were divided into two groups: Group 1 (PG): patients with CHF and periodontitis (*n* = 29; 10 women; 19 men) and Group 2 (NPG): CHF patients with no periodontitis (*n* = 29; 18 women; 11 men). According to the inclusion criteria, 32 patients were initially qualified for the PG, but three patients were older than 80 years and therefore excluded. Thus, two CHF groups with and without periodontitis of equal size were created.

The diagnostic investigations also included the cardiocirculatory, metabolic, and anthropometric data, periodontal status, echocardiography, and laboratory and exercise tests. The following parameters were considered for the present analysis: high-sensitivity C-reactive protein (hsCRP), interleukin-6 (IL6), leukocytes, NT-proBNP, hsTrop T, and blood lipid status. Blood sampling was performed between 8 and 9 a.m. after overnight fasting. Written informed consent was obtained from all participants after they confirmed their complete understanding of the study protocol.

### 2.3. Periodontal Examination

All patients underwent a dental examination by the same experienced dentist. As a method for assessment, the periodontal screening and recording (PSR) was used. The PSR was measured using the WHO probe. The dentition was divided into sextants (S1–S6). The probe was inserted into the sulcus from the oral and vestibular sides and moved once around the tooth at the base of the sulcus (without pressure). The deepest measurement is decisive for the code in the respective sextant. The PSR scale and criteria and criteria are as follows: Code 0 indicates periodontal health, codes 1 and 2 indicate gingivitis, and codes 3 and 4 indicate periodontitis. If a code 3 or 4 is found, a comprehensive examination and treatment are necessary. PSR code 3 is defined as a probing depth ≥ 3.5 mm ≤ 5.5 mm, and PSR code 4 as a probing depth ≥ 5.5 mm. The presence of calculus, gingival inflammation, and periodontal pocket depth was recorded, and the resulting periodontal screening and recording (PSR) value was calculated.

### 2.4. Questionnaires

MEDAS: The Mediterranean Diet Adherence Screener was used to determine patients’ adherence to the Mediterranean diet [40]. It includes 14 questions (Table 1). The evaluation is performed by earning 0 or 1 points (Table 1). If a criterion is not met, 0 points are recorded. A total of 14 points is the maximum.

OHIP-14: A short form, the OHIP-14 [42,43], was used for oral health-related quality-of-life (oral QoL) assessments. The OHIP-14 has 14 items with answers rated on a 5-point scale (from 0 = never to 4 = very often), with a maximum score of 56 to indicate a level of different problems related to oral health over in the last month [43]. The higher the score, the worse is the impact on oral health.

Modified PHQ-9 and PHQ-2: The Patient Health Questionnaire module 9 (PHQ-9) was modified by omitting the last question (criteria nine “thoughts that you would be better off dead or of hurting yourself in some way”). This questionnaire comprised eight questions on depressiveness as a screening instrument for the diagnosis of depression for routine use. Major depression is diagnosed if five or more of the depressive symptom criteria have been present at least “more than half the days” in the past two weeks, and one of the symptoms is a depressed mood or anhedonia. Other depression is diagnosed if 2, 3, or 4 depressive symptoms have been present at least “more than half the days” in the past two weeks, and one of the symptoms is a depressed mood or anhedonia [44]. As a severity measure, the PHQ score of the current study can range from 0 to 24, since each of the eight items can be scored from 0 (not at all), 1 (several days), or 2 (more than half of the days) to 3 (nearly every day) in the last two weeks. Total scores of 5, 10, 15, and 20 represent the cut points for mild, moderate, moderately severe, and severe depression, respectively [44]. The PHQ-2 is an ultra-short form and consists of only the first two items of PHQ-9, concerning loss of interest and depressive mood.

### 2.5. Blood Sampling

Blood samples were collected to assess potentially predictive outcome factors. Ethylenediaminetetraacetic acid plasma was analyzed at each study center, and serum was collected, centrifuged, and stored at minus 80 °C until analysis.

### 2.6. Echocardiography

All patients had a standard transthoracic echocardiographic examination. For a simplified approach, the ejection fraction (EF biplane from a four- and two-chamber view, EF_bip_) was determined for the systolic function and E/A or E/e’ for the diastolic function. Right and left ventricular longitudinal shortenings were measured as the TAPSE (tricuspid annular plane systolic excursion) and lateral MAPSE (lateral mitral annular plane systolic excursion, MAPSE_lat_). Due to the limited study size, no further differentiation of HF with HFrEF or preserved HFpEF was performed.

### 2.7. Incremental Test

An incremental exercise test was performed using a semi-recumbent cycle ergometer with an inclination angle of 35°. The saddle height and pressure point of the pedals were adapted to the individual body proportions of the participants. The feet were firmly fixed to the ergometer pedals. The pedal frequency was set at 60–70 rpm. A permanent decrease below 60 rpm was considered as the load termination. The load started with a resistance of 30 Watts and continued with increments of 10 Watts every minute until exhaustion. Before the start of the test and during every third minute of the test, the systolic (S) and diastolic (D) blood pressure (BP) and subjective stress sensation were measured. Additionally, all parameters were measured in the post-exercise first, third, and fifth minute. The heart rate (HR), electrocardiography, and spirometry data were recorded continuously throughout the test. The discontinuation criteria for cycle ergometry were according to the guidelines of the German Society for Cardiology: Cardiovascular Research.

### 2.8. Statistics

The statistical analyses were performed using GraphPad InStat Software Version 8 (GraphPad Software, La Jolla, CA, USA). All data are presented as the means ± SD. To test for significant differences between the groups and measurements, the Mann–Whitney U-test was used. The Spearman test was used for nonparametric correlation analyses. All tests were two-tailed. A *p*-value < 0.05 was considered significant.

## 3. Results

### 3.1. Summary of Findings

A total of 58 patients (mean age: 66.95 ± 9.40 years; 28 females; 30 males) with CHF were included. They were categorized into the following groups: PG: patients with CHF and periodontitis (PG; *n* = 29; NYHA I [45%], NYHA II [55%]) and NPG: patients with CHF and without periodontitis (NPG; *n* = 29; NYHA I [52%], NYHA II [48%]. Three patients in the PG and two patients in the NPG had diabetes mellitus. Valvular diseases concerned four patients in the PG and seven patients in the NPG.

There were no significant differences between the groups concerning the height, weight, body mass index (BMI), hemoglobin A1c (HbA1c), number of teeth, but there was a significant age difference. According to the group classification by the criterion of periodontal status, there was a significant difference in the mean PSR value between the groups (Table 2). The mean NT pro-BNP and troponin T levels were found to be significantly higher in the PG when compared to the NPG.

No significant differences between the groups were found for the inflammatory parameters hsCRP, IL-6, and leukocytes. Also, the cardiocirculatory and performance parameters as well as the age-related references showed no significant difference between the groups (Table 2). The mean RPP (rate–pressure product) as a reliable index of myocardial oxygen demand was not significantly different among groups as well as the NYHA functional classes. A significantly lower E/A ratio, ejection fraction (EF_bip_), and lateral MAPSE_lat_ were found in the PG (Table 2).

### 3.2. MEDAS

**Figure 1:**
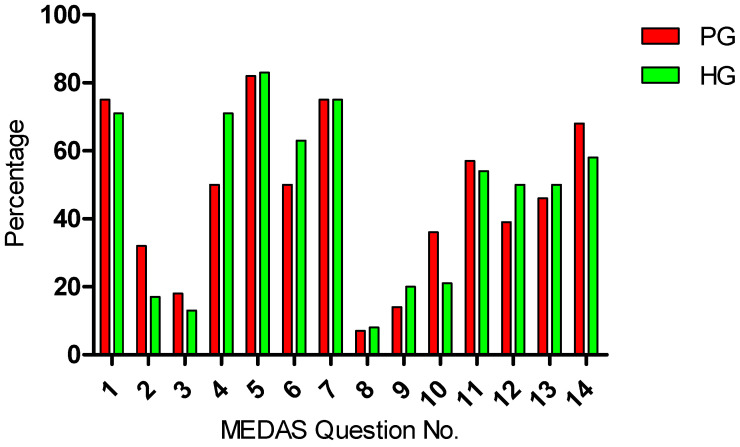
Mediterranean Diet Adherence Screener (MEDAS) responses in detail.

The mean MEDAS score of both groups was almost equal and can be classified as a medium with a score of 6.50 (PG) and 6.54 (NPG) [41]. However, double-digit percentage differences were seen between the groups. Accordingly, the PG consumed less fruits and nuts and more olive oil, fats, and fish in comparison to the NPG. Figure 1 shows the differences in the respective MEDAS answers of both groups.

### 3.3. OHIP-14 and PHQ

The mean OHIP-14 score in the PG was 5.26 ± 7.16 vs. 3.52 ± 4.35 in the NPG (*p* < 0.87). An OHIP-14 score between 14 and 28 was found in four patients (PG) and in one NPG patient (score = 19).

The mean PHQ score of both groups differed insignificantly (5.04 ± 4.22 in PG vs. 4.70 ± 3.61 in NPG). Major depression is diagnosed if five or more of the depressive symptom criteria have been present at least “more than half the days” in the past two weeks, and one of the symptoms is a depressed mood or anhedonia. This concerned one patient in both groups. Other depression is diagnosed if 2, 3, or 4 depressive symptoms have been present at least “more than half the days” in the past two weeks, and one of the symptoms is a depressed mood or anhedonia. This concerned four patients in the PG and none in the NPG. The highest PHQ scores in the PG were 11, 12, and 17 vs. 10 and 17 in the NPG.

The PHQ-2 focuses only on the first two items of the PHQ-9 according to the DSM-IV (*Diagnostic and Statistical Manual of Mental Disorders IV*: loss of interest and a depressed mood. One of these two items was true for five patients in the PG and one patient in the NPG. In both groups, one patient answered both questions positively.

### 3.4. Correlation Analysis

A systematic correlation analysis (Spearman test) of the mean PSR value within the PG revealed a significant correlation with the tooth count (*p* < 0.004; *r* = −0.55).

Additionally, the data of the total sample were analyzed in a correlation matrix. This revealed a correlation between the age and mean PSR value (*p* < 0.0008; *r* = 0.43), NT-proBNP (*p* < 0.02; *r* = 0.31), and hsTropT (*p* < 0.0001; *r* = 0.59) as well as between the hsTropT and NT-proBNP values (*p* < 0.0025; *r* = 0.40). The mean PSR value correlated significantly with the hsTropT levels (*p* < 0.017; *r* = 0.42). The PSR and BMI correlated significantly (*p* < 0.03; *r* = 0.29), as well as the BMI with the total cholesterol (*p* < 0.04; r = −0.28) and WC (*p* < 0.0001; *r* = 0.80). The EF biplane correlated significantly with the MAPSE_lat_ (*p* < 0.0165; *r* = 0.32).

No correlation between the mean PSR and the NT-proBNP, total cholesterol, WC, triglycerides, LDL and HDL, EF, E/A, or MAPSE_lat_ was seen. There was also no correlation between the age and E/A, EF, or MAPSE_lat_.

## 4. Discussion

### 4.1. Inflammatory Parameters

Chronic infections and inflammation in the periodontal tissues are typical signs of periodontitis. They are the result of an interaction between tooth-associated microbial biofilms and host defenses. Periodontal pathogens (*Porphyromonas gingivalis*, *Tannerella forsythia*, *Prevotella intermedia*, *Fusobacterium nucleatum*, *Treponema denticola*, and *Actinobacillus actinomycetem comitans*) can influence the local and systemic immune and inflammatory responses [45]. They produce various endotoxins that can interact with toll-like receptors on the immune cell surface and activate signal transduction pathways in the immune system. This leads to the production of proinflammatory cytokines (e.g., IL-6) that can induce the hepatic production of acute-phase proteins including CRP [22,46]. Periodontal disease has been found to be associated with elevated CRP levels [47]. Increased levels were also found [48,49] when compared to healthy controls [45]. PD may induce a biological burden of endotoxins and inflammatory cytokines that trigger and exacerbate inflammation, atherogenesis, and the development of cardiovascular disease [50]. A recent study showed that the periodontal status was associated with CHF and unfavorable changes in CRP and increased NT-proBNP values [51]. This is in line with a cross-sectional analysis of NHANES III [23], showing that PD was an independent risk factor for the presence of CHF.

Inflammation is originally a healing and protective reaction. However, if this process is uncontrolled or chronically systemic, it can also cause tissue damage. Inflammatory cytokines exert direct effects on cardiac and vascular cells, predisposing the development of CHF [52]. IL-6 promotes myocyte hypertrophy and increases myocardial stiffness by decreasing the phosphorylation of titin [53]. Studies showed that IL-6 and CRP are strongly and independently associated with all new cases of CHF [54,55], and the 5-year risk of developing CHF augments by 68% per tertile increase in IL-6 levels [56]. Schulze-Spate et al. [24] showed an association between CHF and periodontitis, indicating that a more severe periodontitis is related to increased turnover markers like inflammatory mediators and cytokines in patients with CHF.

C-reactive protein and interleukin-6 [57] are associated with the CHF prognosis. Thus, these serum biomarkers may be predictors of a worse NYHA functional class [58] or reduced left ventricular function [59], and IL-6 may act as an adjunct for risk stratification [60].

However, the current study failed to show higher hsCRP and IL-6 serum levels in the PG vs. NPG. Both groups showed almost equal CRP levels (mean 2.1 mg/L; PG vs. NPG: n = 12/14 low, 12/9 moderate, and 5/6 high risk). In healthy individuals, the CRP levels were found to be ≤0.3 mg/L [45]. When used for cardiac risk stratification, hsCRP levels less than 1 mg/L are considered low risk. Levels between 1 mg/L and 3 mg/L are considered moderate risk, and levels higher than 3 mg/L are considered high risk for the development of cardiovascular disease [61,62]. Despite undiagnosed periodontitis, the CRP values of the NPG were above this value for healthy individuals. However, it is important to consider that the mean CRP levels of both groups were within the reference range of moderate risk.

The results of the current study showed significantly higher hsTropT and NT-proBNP values in the PG. A significant correlation between the age and mean PSR value, NT-proBNP, and hsTropT was identified. This indirectly points to an inner relationship, and at least no exclusion of a cause–effect relationship can be made. The disease severity increases with age as well as the CHF stage and NT-proBNP levels. A previous study showed also that patients with chronic HF who had severe periodontitis were older than those who had gingivitis [2]. A recent review found elderly individuals to be more vulnerable to periodontitis [63]. Studies revealed a compromised bone metabolism in those with HF [9], and patients with severe CHF showed increased serum bone markers [10]. Bone catabolism and pro-inflammatory cytokines can enhance periodontal disease [64]. In the current study, no correlation was found between PSR and NT-proBNP. This is in line with another study in patients with CHF, which showed no correlation between the periodontitis severity and NT-proBNP levels [2]. However, the occurrence of increased PSR together with an older age and increased cardiac risk parameters does not exclude an association between periodontitis and CHF.

The overall results show that the generally assumed cascade of inflammation and PD as well as inflammation and CHF cannot be confirmed in the current study. Age apparently was the only independent risk factor. PD seems to get worse with the progression of CHF and age. Therefore, a consistent PD prevention regimen or treatment in NYHA classes I and II seems to be important, as the inflammatory status and development can still be positively influenced at that time. A recent study shows that professional dental cleaning and the number of missing teeth were associated with CHF [25]. In the current PG, a higher PSR value correlated with a lower tooth count. Apart from age, this can be explained by a possible tooth loss due to periodontal disease.

The current results demonstrate that it is important to assess and control the oral health status when dealing with patients with CHF and measure the serum cardiac inflammatory biomarkers. Improved oral hygiene was associated with a decreased risk of atrial fibrillation and heart failure [25]. In any case of oral inflammation or periodontal disease, the physicians must refer the patients to a dentist for oral and periodontal treatment. Maintaining good oral health has an essential impact on the inflammatory burden in patients with heart failure, especially in older patients.

### 4.2. PSR and BMI

In the current study, the BMI was associated with the WC, total cholesterol levels, and PSR. An increased odds ratio of periodontitis in individuals with obesity was found in a recent systematic review and meta-analysis: among the 29 included observational studies, 17 showed significantly increased and 3 showed significantly decreased odds ratios, whereas 9 studies found no association [63]. The BMI measures the excess weight rather than body fat and is influenced by age, gender, and muscle mass [65]. Further studies reported that an increased BMI, waist circumference (WC), body fat, and serum lipid levels are associated with periodontitis development [46,66,67,68]. A BMI is frequently used but does not represent the body fat distribution. Abdominal obesity, measured by the waist circumference, has a higher risk for CVD. Abdominal adipose tissue secretes higher levels of cytokines, adipokines, and leptin than subcutaneous adipose tissue [66], resulting in pro-inflammatory effects. Obesity can enhance chronic inflammatory diseases (e.g., diabetes and atherosclerosis) [69] that are induced by adipokines [70]. A body mass index over 30 is considered obese [65]. Of 58 CHF patients in the current study, only 12 (20.7%) had a BMI ≥30 kg/m^2^. No correlation was found between the WC and serum inflammatory markers (CRP and IL-6). The BMI correlated significantly with the mean PSR values. Body weight control may be a modifiable risk factor to prevent or alter the periodontitis status and the inflammatory burden. Therefore, weight management and registration are important to maintain periodontal health and reduce the risk of CHF at the same time. A causal relationship between the BMI, obesity, and periodontitis can be seen in common parts of the pathophysiology such as inflammation, age, and diabetes mellitus [66]. Diabetes mellitus is considered a risk factor for vascular complications and delayed wound healing. Researchers also presume that oral vascular changes are a predisposing factor for PD. Conversely, periodontitis aggravates the prevalence and complications of diabetes [71]. In the current study, five patients (three patients in the PG and two in NPG) had diabetes mellitus. Due to the small sample size, we could not further analyze this impact on our results. However, the distribution was almost the same across both groups, and the mean HbA1c values showed no significant differences.

### 4.3. MEDAS

The Mediterranean diet’s final adherence could be classified as a medium with a score of 6.50 (PG) and 6.54 (NPG) [41]. Olive oil and fat consumption were higher and nut and fruit intake were lower in the PG, and this might also have had an influence on periodontitis. It has been observed that the lipid composition of cell membranes and blood lipid profile can be modified through the diet [72,73], which has been linked to susceptibility to oxidative damage [74]. Likewise, the response against certain bacterial products can also be modulated by the membrane lipid profile [75]. For these reasons, some modifications in the dietary patterns to affect lipid profiles could be interesting for both preventing periodontal diseases and improving periodontal health [76,77]. Polyunsaturated fatty acids (e.g., omega-3 fatty acids) also present in nuts are reported to have a beneficial effect on periodontal health [78]. There is quite a lot of information in favor of a positive role of omega-3 fatty acids due to their antioxidant and immunomodulatory effects. On the other hand, saturated fat-rich diets increase oxidative stress as well as the intensity and duration of inflammatory processes [77]. The type of fat in the diet is related to both general health and periodontal health [79].

Polyphenol antioxidants (e.g., flavonoids, phenolic acids, and carotenoids) contained in fruits (e.g., dark berries) and vegetables (e.g., dark leafy greens) have been claimed as potentially active against oral and dental diseases [80,81]. Recent studies showed that reduced levels of antioxidant micronutrients compromise periodontal health in the cases of gastrointestinal disorders, poor diet, or poor lifestyle [82]. A healthy diet should contain several antioxidants in the form of micronutrients, such as vitamins A, C, E, and glutathione, which all have an impact on periodontal health [83,84]. Vitamin A is a fat-soluble vitamin and has been used to supplement periodontal treatments [83,85] and showed slight improvements. Dietary sources of vitamin A include eggs, carrots, liver, sweet potato, broccoli, and leaf vegetables. A prospective cohort study reported that low serum B12 levels were associated with a worsening of the periodontal status [86]. Vitamin C is required for collagen synthesis [87], and it also enhances iron absorption [88]. The likelihood of periodontal disease has been shown to be 20% greater in those with a low vitamin C intake [89]. The health benefit of phenolic consumption derives from the synergistic activity of the bioactive compounds and other nutrients contained in fruits, vegetables, and whole grains. Several studies have demonstrated a correlation between reactive oxygen species (ROS) and periodontal disease activity [90]. Antioxidants may overcome the ROS-mediated periodontal inflammation [91,92].

### 4.4. OHIP-14 and Modified PHQ-9

Oral health and depression can affect general health, treatment adherence, and the quality of life in patients with CHF. Therefore, it was one aim of the study to characterize the oral health and psychological burden in patients with CHF.

The questionnaires showed no significant differences. The overall OHIP-14 score averaged 3.52 (±4.35) in the NPG, indicating very little impairment in the oral health-related quality of life (QoL). In addition, the PG had a higher mean OHIP score of 5.26 (±7.16). The mean PHQ score in the PG was 5.04 ± 4.22 vs. 4.70 ± 3.61 in the NPG. PHQ scores less than 5 almost always signified the absence of a depressive disorder; scores of 5 to 9 predominantly represented patients with either no depression or subthreshold depression; scores of 10 to 14 represented a spectrum of patients; scores of 15 or greater usually indicated major depression [44]. The mean PHQ score in the PG was slightly higher than 5, indicating minimal symptoms and proposing a treatment recommendation such as support, call in case of aggravation, and return in one month [93]. A depression diagnosis that warrants treatment or a treatment change needs at least one of the first two questions endorsed as positive (little pleasure, feeling depressed), indicating the symptom has been present more than half of the time in the past two weeks [44]. The PHQ-2 focuses on the two main criteria of major depression according to the DSM-IV (*Diagnostic and Statistical Manual of Mental Disorders IV*): loss of interest and a depressed mood. This criterion was true for five patients in the PG and only one patient in the NPG. In both groups, one patient answered the first two questions positively. In the PG, four patients reported 2, 3, or 4 depressive symptoms at least “more than half the days” in the past two weeks, and one of the symptoms was a depressed mood (first item) or anhedonia (second item). This tends to indicate an increased depressive mood in the PG. Studies showed that depressed patients have poorer oral health with higher caries incidence, worse periodontal status, and increased tooth loss, so they require more intensive dental care [94,95]. Poor oral health can affect nutrition, appearance, self-esteem, and socialization. Overall, these factors can also influence the patient´s wellbeing and (adherence to) CHF treatment. Conversely, depression can also affect all these factors.

### 4.5. Cardiocirculatory Parameters

Overall, there were no significant differences in the circulatory and performance parameters between the two groups. However, significant differences were calculated for the echocardiographic systolic function (EF_bip_, MAPSE_lat_) and diastolic function (E/A), with lower values for the PG. MAPSE_lat_ values in the PG were also well below the age-related reference values [96]. Yan et al. [97] showed that the incidence of heart failure in patients with moderate/severe periodontitis was markedly higher compared to no or mild periodontitis. Walther et al. [98] reported an association between severe periodontitis and mid-range-reduced EF, but not with HF in general or echocardiographic variables. The fact that in the current study the maximum ergometry values were similar in both groups may point to the hypothesis that the lower echocardiographic systolic and diastolic function values as well as the higher cardiac-specific laboratory parameters are more likely attributable to periodontitis than to age.

### 4.6. Limitations

A limitation of the study is the small sample size due to strict recruiting guidelines and characteristics for group assignment. This may affect the generalizability of the results. Of 132 CHF patients from the same area of residence screened for periodontal health, only 58 were found eligible for the two study groups focusing on periodontitis. Therefore, the study assessed and compared the data among CHF patients and coexistent periodontitis, with no age-matched CHF controls (70.69 ± 6.6 vs. 63.21 ± 10.4 years). Only patients with NYHA classes I and II were found in the present study. Patients with pronounced heart failure and periodontitis may be more likely to have higher levels of inflammatory serum markers. However, only a small number of such patients applied for the study and were not included due to certain exclusion criteria. This may affect the generalizability of the results to more severe CHF. Residual confounding also remains in our analysis, as the results were adjusted only for age and BMI. Due to the small patient collective, the impacts of other factors, especially of comorbidities, were not further analyzed in our results. Diabetes mellitus was found as a comorbidity in three patients in the PG and two patients in NPG. Valvular disease was detected in four and seven cases, respectively. Due to the small group sizes, it was not possible to specify the CHF subtypes in a subgroup of patients, which could be another interesting factor. The studied patient collective was mostly married, with a higher level of education. Thus, a better access to or interest in health care information and a better understanding of CHF treatment options with the expected consequences may be assumed for the total current study collective.

Future studies are needed for a better understanding of the possible mechanisms of periodontitis effects on CHF development. Randomized control trials are necessary to determine how periodontal treatments affect cardiac and systemic inflammatory markers of CHF. Further, more research investigating the potentials of periodontal therapies and prophylaxis as prevention strategies to minimize the CHF burden is warranted.

## 5. Conclusions

The current cross-sectional study showed the following: (1) The serum levels of hsCRP, IL6, and leukocytes were not higher in CHF patients with periodontitis. (2) The serum levels of NT-proBNP and TropT were significantly higher in CHF patients with periodontitis, and the left ventricular systolic and diastolic function parameters were significantly lower. (3) Periodontitis correlated with age and BMI. Identifying low-grade chronic systemic inflammation, e.g., periodontitis, may be promising for treatments. Our observations, linking the periodontal status to NT-proBNP and hsTropT levels, joins in the hypothesis that chronical periodontal inflammation may contribute to cardiac abnormalities. This highlights the need for further research to investigate the potential association and the potential of periodontal treatment to reduce the risk of developing or worsening CHF.

Prospective longitudinal studies are needed to confirm the observed associations and to clarify a possible causal relationship between PD and CHF. Periodontitis may be a modifiable risk factor for CHF. If future studies support the evidence of a causal relationship, the impact could be significant given the high prevalence of treatable periodontitis and the burden of CHF in our aging society. Already at the current level of emerging studies, it is important to consider the oral health status of patients when dealing with heart-related diseases. The treatment of oral infections may help to treat heart diseases and to control the inflammatory burden. In any case of suspected oral inflammation or periodontal disease, the physicians must refer the patients to a dentist for oral tissue treatments. Overall, good oral hygiene practices, oral health promotion, routine periodic assessments every six months, educational interventions, and personalized dental care instructions are necessary for CHF patients. A sustainable management of oral hygiene, weight control, and physical fitness may be an efficient way to decrease heart failure symptoms and periodontitis.

## Figures and Tables

**Table 1:** MEDAS questionnaire [41].

Questions	Criteria for 1 Point
1. Do you use olive oil as a main culinary fat?	Yes
2. How much olive oil do you consume in a given day (including oil used for frying, salads, out-of-house meals, etc.)?	≥4 tablespoons
3. How many vegetable servings do you consume per day? (1 serving = 80 g)	≥5 or ≥3 portions of raw vegetables or salad
4. How many fruit units (including natural fruit juices) do you consume per day?	≥3
5. How many servings of red meat, hamburger, or meat products (ham, sausage, etc.) do you consume per day? (1 serving = 100–150 g)	<1
6. How many servings of butter, margarine, or cream do you consume per day? (1 serving = 12 g/1 tablespoon)	<1
7. How many sweet or carbonated beverages do you drink per day?	<1
8. How many glasses of wine do you drink per week?	≥10 glasses (0.25 L = 1 glass)
9. How many servings of legumes do you consume per week? (1 serving = 150 g)	≥3
10. How many servings of fish or shellfish do you consume per week? (1 serving = 100–150 g of fish or 200 g of shellfish)	≥3
11. How many times per week do you consume commercial sweets or pastries (not homemade), such as cakes, cookies, biscuits, or custard?	<3
12. How many servings of nuts (including peanuts) do you consume per week? (1 serving = 30 g)	≥3
13. Do you preferentially consume chicken, turkey, or rabbit meat instead of veal, pork, hamburger, or sausage?	Yes
14. How many times per week do you consume vegetables, pasta, rice, or other dishes seasoned with sofrito (sauce made with tomato and onion, leek, or garlic and simmered with olive oil)?	≥2

**Table 2:** Anthropometric data, dental status, and cardiac, inflammatory, lipid, and cardiocirculatory parameters of both groups.

Parameter	Reference	PG *n* = 29	NPG *n* = 29	*p*-Value
Age [years]		70.69 (6.6)	63.21 (10.4)	<0.004
Height [cm]		171.5 (8.3)	173.4 (10.6)	n.s.
Weight [kg]		82.2 (15.1)	78.4 (15.6)	n.s.
BMI [kg/m^2^]		27.8 (3.9)	26.0 (4.3)	n.s.
WC [cm]		100.1 (14.0)	92.6 (13.5)	n.s.
Number of teeth		23.7 (6.3)	25.1 (4.1)	n.s.
Mean PSR		3.2 (0.7)	0.3 (0.3)	<0.0001
NT-proBNP [pg/mL]	<125	642 (807)	341 (256)	<0.023
hsTropT [pg/mL]	<14	11.7 (5.0)	7.1 (4.7)	<0.001
CRP [mg/L]	Low < 1 Moderate 1–3 High > 3	2.1 (2.1)	2.1 (2.3)	n.s.
IL-6 [pg/mL]	<7	3.8 (2.4)	3.5 (2.2)	n.s.
HbA1c [%]	<6.5	5.9 (1.0)	5.6 (0.5)	n.s.
Leukocytes [10^4^/µL]	3.5–9.8	6.4 (2.1)	6.1 (1.5)	n.s.
Total cholesterol [mmol/L]	<5.2	4.66 (1.1)	4.66 (1.38)	n.s.
Triglyceride [mmol/L]	<1.7	1.19 (0.44)	1.28 (0.60)	n.s.
LDL [mmol/L]	<4.2	2.86 (0.85)	2.80 (1.12)	n.s.
HDL [mmol/L]	>1.03	1.19 (0.44)	1.28 (0.60)	n.s.
EF_bip_ [%]	>51	48.42 (9.17)	53.38 (6.39)	<0.022
E/A	>1.1	0.88 (0.24)	1.07 (0.33)	<0.040
E/e’	<14	10.13 (4.49)	9.04 (3.47)	n.s.
TAPSE [cm]	≥1.6	2.00 (0.37)	2.10 (0.34)	n.s.
MAPSE_lat_ [cm]	≥1.4	1.17 (0.23)	1.34 (0.19)	<0.005
SBP [mmHg]		143.3 (14.5)	138.7 (15.6)	n.s.
DBP [mmHg]		84.0 (9.5)	84.6 (8.1)	n.s.
SBP ET [mmHg]		202.2 (30.4)	194.4 (28.3)	n.s.
DBP ET [mmHg]		92.0 (11.3)	91.6 (11.2)	n.s.
HR ET [bpm]		133.8 (18.9)	141.7 (23.2)	n.s.
% reference HRmax [bpm]		89.2 (13.4)	90.1 (14.6)	n.s.
Pmax [Watt]		111.7 (37.8)	117.9 (50.9)	n.s.
% reference Pmax [Watt]		82.0 (27.0)	85.4 (31.7)	n.s.
RPPmax [mmHg × bpm × 100^−1^]		268.85 (61.97)	274.11 (67.03)	n.s.
RPP/Watt [mmHg × bpm × 100^−1^ × Pmax-1]		2.50 (0.72)	2.52 (0.76)	n.s.

Body mass index (BMI), waist circumference (WC), periodontal screening and recording (PSR), N-terminal pro b-type natriuretic peptide (NT-proBNP), high-sensitivity troponin T (hsTropT), C-reactive protein (CRP), interleukin-6 (IL-6), hemoglobin A1c (HbA1c), low density lipoprotein (LDL), high density lipoprotein (HDL), ejection fraction (EF), ratio of peak velocity blood flow from left ventricular relaxation in early diastole to peak velocity flow in late diastole caused by atrial contraction (E/A), ratio of early diastolic mitral inflow velocity to early diastolic mitral annulus velocity (E/e’), tricuspid annular plane systolic excursion (TAPSE), lateral mitral annular plane systolic excursion (MAPSE_lat_), systolic and diastolic blood pressures at rest (SBP and DBP) and at maximum load of exercise test, heart rate (HR), as well as the percentage of the age-related maximum reference values of HR (220 minus age) and performance (P); maximum rate–pressure product (RPP; HR × SBP × 100^−1^), also calculated per Watt. Values are given as means, with the SD in brackets; n.s. = not significant.

## Data Availability

Further information on the data set and materials is available from the corresponding author upon reasonable request.
